# Rapid RNA analysis of individual *Caenorhabditis elegans*^☆^

**DOI:** 10.1016/j.mex.2015.02.002

**Published:** 2015-02-07

**Authors:** Kien Ly, Suzanne J. Reid, Russell G. Snell

**Affiliations:** School of Biological Sciences, The University of Auckland, Auckland 1010, New Zealand

**Keywords:** Rapid single worm RT-qPCR, *C. elegans*, RNA, RT-qPCR

## Abstract

Traditional RNA extraction methods rely on the use of hazardous chemicals such as phenol, chloroform, guanidinium thiocyanate to disrupt cells and inactivate RNAse simultaneously. RNA isolation from *Caenorhabditis elegans* presents another challenge due to its tough cuticle, therefore several repeated freeze–thaw cycles may be needed to disrupt the cuticle before the cell contents are released. In addition, a large number of animals are required for successful RNA isolation. To overcome these issues, we have developed a simple and efficient method using proteinase K and a brief heat treatment to release RNA of quality suitable for quantitative PCR analysis.The benefits of the method are:

• Faster and safer compared to conventional RNA extraction methods
• Released RNA can be used directly for cDNA synthesis without purification
• As little as a single worm is sufficient

## Graphical abstract

**Figure:**
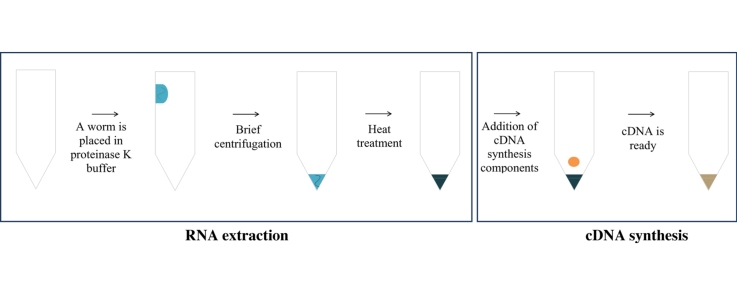

## Method

*Caenorhabditis elegans* were cultured under standard laboratory condition [6]. Wild type N2 *C. elegans* was used and propagated at 20 °C in NGM (nematode growth medium) plates seeded with *Escherichia coli* OP50. The *C. elegans* and *E. coli* were obtained from Caenorhabditis Genetics Center.

## RNA extraction, genomic DNA digestion, cDNA synthesis and RT-qPCR

A single worm was picked from an NGM plate using an eyelash pick and transferred to a drop of ∼20 μL of H_2_O to remove bacteria. The cleaned worm was transferred using the eyelash pick into 1 μL of worm lysis buffer (5 mM Tris pH 8.0 (Sigma–Aldrich), 0.5% Triton X-100 (Sigma–Aldrich), 0.5% Tween 20 (Bio-rad), 0.25 mM EDTA (Merck) and 1 mg/mL proteinase K (Roche)) on the wall of a 0.2 mL PCR tube. The tube was briefly centrifuged to bring the worm to the bottom of the tube and then incubated at 65 °C for 10 min in a thermocycler (Eppendorf). To inactivate proteinase K, the tube was heated to 85 °C for 1 min and then immediately cooled on ice. The worm lysate was used immediately for cDNA synthesis or stored below −70 °C. A single adult worm typically contained ∼35 ng total RNA, measured using Qubit RNA HS Assay kit (Life Technologies).

cDNA synthesis was performed using Maxima H Minus cDNA synthesis kit (Thermo Fisher) as described in the manufacturer’s manual. One microliter of cDNA synthesis mix was added to the worm lysate. The final mix contains 1× supplier-provided buffer, 0.5 mM each dNTP, 5 μM random hexamer, heat labile dsDNAse 1 unit/μL RNAse inhibitor and 20 unit/μL reverse transcriptase. The tube was briefly centrifuged, mixed, and incubated at 25 °C for 10 min, followed by 55 °C for 30 min and a final 85 °C for 5 min. The cDNA was diluted to 25 μL with H_2_O, used immediately or stored below −70 °C.

Quantitative PCR was performed using Roche LightCycler^®^ 480 System. Each 10-μL reaction contained 0.5 μM each of forward and reverse primers, 0.1 μM hydrolysis probe, 1× Probes Master, and 2.5 μL of diluted cDNA. The primers for the quantification of the reference gene *act-1* were manufactured by IDT and the sequences are: act1f (ACGCCAACACTGTTCTTTCC), act1r (GATGATCTTGATCTTCATGGTTGA), in 5′ → 3′ direction.

As a comparison, RNA extraction from worms was also performed using Trizol (Life Technologies) [3]. Briefly, 0.5 mL of Trizol was added to worms in a 1.5 mL tube and incubated at RT for 5 min. Chloroform (0.1 mL) was added and the tube was shaken vigorously for 15 s. The tube was incubated at room temperature for 2–3 min and then centrifuged at 12,000 × *g* for 15 min at 4 °C. The aqueous phase was transferred into a new tube and 0.25 mL of isopropanol was added. The tube was incubated at room temperature for 10 min and then centrifuged at 12,000 × *g* for 15 min at 4 °C. The supernatant was removed and the tube was washed with 1 mL of ice cold 70% (v/v) ethanol. The tube was air dried and resuspended in RNAse-free H_2_O. When extracting from a single worm, the precipitate was finally resuspended in 1 μL of H_2_O; when extracting from 100 worms, 100 μL of H_2_O was used. One microliter of purified RNA was used for cDNA synthesis (as described above).

**Fig. 1:**
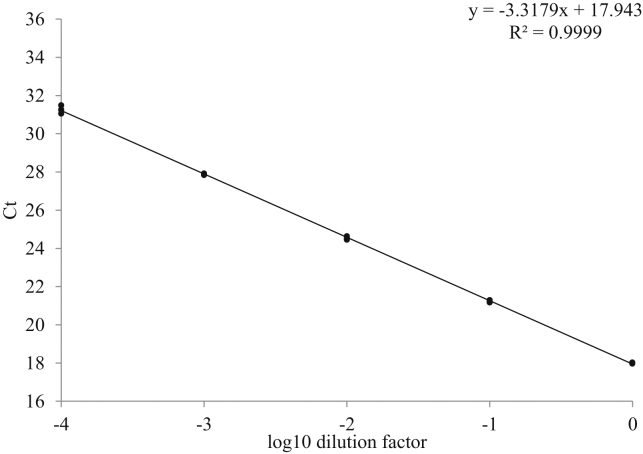
Standard curve for *act-1*. Efficiency is 100.167%, calculated based on the formula: *E* = −1 + 10(−1/slope). The slope is −3.3179. The *R*^2^ is 0.9999.

We demonstrate, using *act-1* as a reference gene and absolute threshold cycle (*C_t_*) values as an indirect measurement, that RNA is released from animals of all developmental stages from eggs to adults (Table 1). The *C_t_* values decreased as expected from eggs to adults, corresponding to an increase in RNA amount. We found that as little as a single egg could produce *C_t_* values within a useful working range. In addition, cDNA produced from each animal treatment (2 μL) is normally diluted to 25 μL and only 2.5 μL is used for each qPCR reaction, thus a single animal is sufficient for 10 qPCR. For a single adult worm, this resulted in a *C_t_* value of 18.9 ± 0.28 for the *act-1* transcript. The worm lysate did not inhibit cDNA synthesis and qPCR as the amplification efficiency for *act-1* was 100.2% (Fig. 1). As expected increasing the number of animals in a lysate from 1 to 4, correspondingly decreased the *C_t_* value. Therefore, it is possible to adjust several parameters (dilution factor, number of animals) to obtain *C_t_* values suitable for different genes. In contrast, attempts to purify RNA from a single adult worm using Trizol were unsuccessful; no *C_t_* value could be computed. When 100 adults were used in the Trizol method, sufficient RNA could be isolated as expected.

## Polymerase chain reaction for the amplification of *ttn-1* transcript

RNA exposed to heat and potential nucleases can degrade rapidly. While RNA degradation can be accounted for in qPCR with appropriate controls [2], other applications such as cDNA cloning or transcriptome sequencing require relatively intact RNA. To assess if intact transcripts could be isolated from a single proteinase K digested worm, we amplified fragments of the *ttn-1* cDNA.

cDNA from a single digested adult worm (1 μL) was used as a template in a 10 μL reaction containing 1× supplier-provided buffer, 0.25 μM each of forward and reverse primer, 0.2 mM each dNTP and 0.2 unit of polymerase (Phusion DNA polymerase, Thermo Fisher). The tube was heated at 98 °C for 1 min, followed by 30 cycles of 98 °C for 5 s, 65 °C for 15 s, 72 °C for 5 min. The PCR products were resolved on 0.8% agarose gel in 1 × TBE. Primers sequences are: ttnf (AGTGCAAGTGGTCACGCAAC), ttnr1 (TGAGGCTTTTACCGGGACTT), ttnr2 (TGTGGCTGCTGGGTAACAAT), ttnr3 (TCCAGTTGAATTGGCAGTTGG), in 5′ → 3′ direction.

**Fig. 2:**
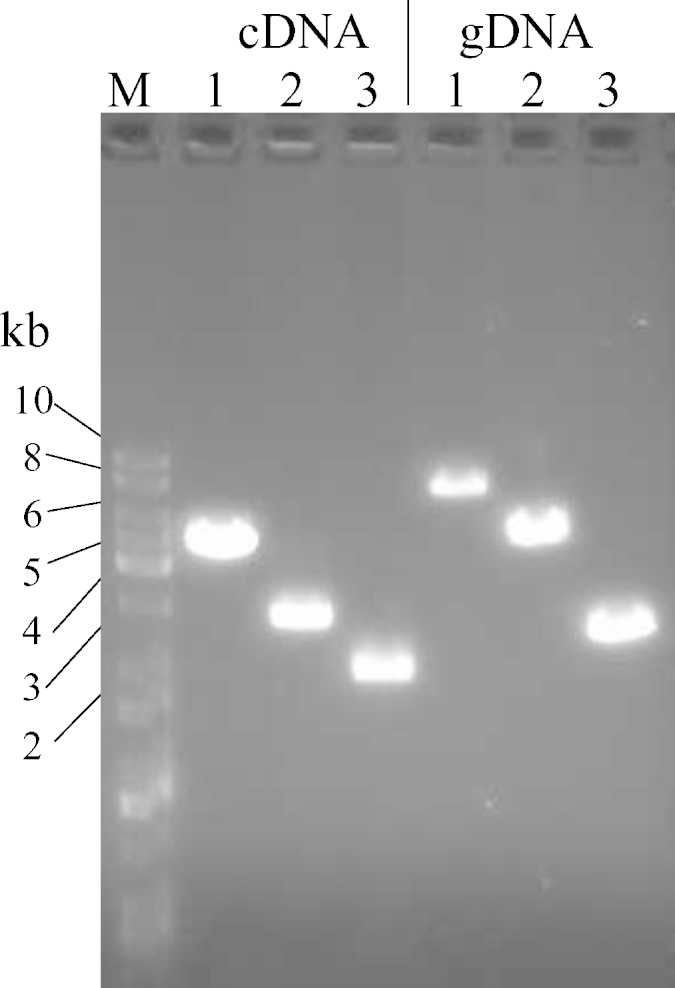
DNA electrophoresis of PCR products from cDNA and gDNA using 3 different sets of primers. M: DNA ladder (Universal Ladder, Kapabiosystems); 1: primer ttnf + ttnr1; 2: primer ttnf + ttnr2; 3: primer ttnf + ttnr3.

Fig. 2 shows that cDNA of at least ∼4 kb was successfully amplified. Three primer pairs sharing a forward primer, with reverse primers located in exon 1–3, amplified fragments of 7.5, 5.0 and 2.5 kb, respectively, from genomic DNA. The amplicons from cDNA with the same three primer pairs generated products of ∼4.5, ∼3.0 and ∼2.0 kb in length, respectively. Thus, this method results in RNA of sufficient quality for not only routine qPCR but also for cloning mRNA transcripts.

## Heat shock mRNA measurement using single animals

**Fig. 3:**
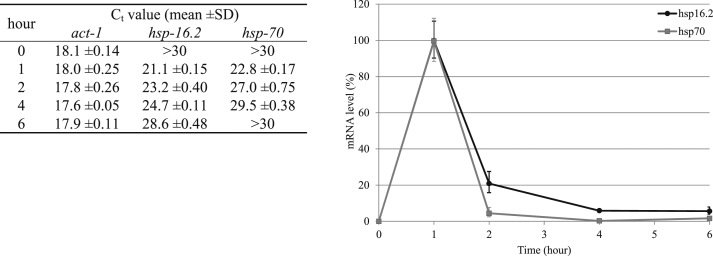
Changes in heat shock mRNA levels (*hsp-16.2* and *hsp-70*, normalized to *act-1*) in *C. elegans* after heat treatment. The animals were raised at 20 °C, upshifted to 30 °C for 1 h and then returned to 20 °C. Error bar: standard deviation; *n* ≥ 5.

To exemplify the usefulness of this method, we measured the expression levels of two heat-shock protein transcripts (*hsp-16.2* and *hsp-70*) from single animals after induction via exposure to high temperature (Fig. 3).

Age-synchronized *C. elegans* was grown on NGM plates at 20 °C until first day adult stage. Immediately before heat shock, animals were collected (time point 0) and treated with proteinase K. The plate was then moved to a dry incubator at 30 °C. After 1 h, the plate was returned to 20 °C and the animals were collected (time point 1). After 1, 3 and 5 h at 20 °C, animals were collected (time point 2, 4, and 6, respectively). cDNA synthesis and qPCR was carried out as above. Fold changes in the two heat shock transcripts (*hsp-16.2* and *hsp-70*) were calculated using the 2^−ΔΔCt^ formula, with *act-1* as reference gene and assuming an amplification efficiency of 100%. The primers are: hsp16f (-TGCAGAATCTCTCCATCTGAGT), hsp16r (TGGTTTAAACTGTGAGACGTTGA), hsp70f (CGGTATTTATCAAAATGGAAAGGTT), hsp70r (TACGAGCGGCTTGATCTTTT), in 5′ → 3′ direction.

At the normal incubation temperature of 20 °C, the levels of the two transcripts were extremely low (*C_t_* values > 30), as expected (Fig. 3). After 1 h of incubation at 30 °C, the level of the two transcripts increased dramatically (*C_t_* values decreased to below 23). Once the animals were returned to 20 °C, the levels of the two transcripts decreased (*C_t_* values gradually increased) and approached the pre heat-shock levels. This rapid alteration in expression level is consistent with other researchers’ findings [5].

In summary, we have developed a simple and efficient method for releasing RNA from as little as a single *C. elegans* which is not possible or very difficult with the widely used Trizol method. Without further purification, the RNA can be used successfully in routine qPCR. This method can dramatically cut down time, reagents and number of animals needed for quantitative analysis of gene expression.

## Additional information

### Background

Transcription analysis is an important step to understanding a physiological state and the analysis of multiple individual animals is likely to provide more insight than a potentially heterogeneous population. A major limitation in the use of *C. elegans* is the inability to extract usable quantities of high quality RNA from individual animals. Due to RNA’s inherent instability and the ubiquitous presence of RNAse most extraction protocols utilize harsh denaturants such as guanidine salt, phenol, and chloroform to rapidly solubilize cells or tissues and irreversibly deactivate RNAse [1]. RNA from *C. elegans* can be purified by phenol/chloroform extraction but requires additional pre-treatment. Typically, the worms are frozen in liquid nitrogen and ground to fine powder before being solubilized in the denaturants [3]. This extra step helps to rupture the worm’s resilient cuticle [4] and release cellular contents effectively. Even though these procedures have been used successfully for isolating RNA from *C. elegans* for many years, there are several drawbacks. A reasonably large number of animals are needed to provide RNA concentration high enough for successful precipitation. Also the chaotropes and organic solvents are highly hazardous. Moreover, the extraction process can be arduous particularly when dealing with a large number of samples. Many cell types from other organisms are readily lysed by mild detergents (or proprietary solutions from commercial kits) and the released RNA can then be used directly in downstream applications. However, the cuticle of *C. elegans* provides a tough barrier resistant to lysis by most solutions. To overcome this, we reported here a simple procedure that can effectively disintegrate *C. elegans* to release RNA ready for use in less than 15 min from one or more animals and without the use of hazardous chemicals. The approach enables population heterogeneity to be examined as this information is lost when extracting from groups of animals. One can analyse multiple individual animals to quantify the variation or to select individual lines with desired phenotypes. One example of this utility is that after microinjection, individual adults are allowed to lay eggs then expression levels of the transgenes are assessed in parents individually. Lines with low or high levels of expressions can then be established. This method will enable high throughput single animal screening.

## Figures and Tables

**Table 1:** Absolute threshold cycles for *act-1* transcript RNA from animals of various stages was either extracted with the proteinase K + heat treatment or Trizol and converted to cDNA. The amounts of cDNA were quantified by qPCR and the *C_t_* values (±SD) (*n* ≥ 6, biological replicates) were used as an indirect measurement of RNA abundance. Extraction of RNA from a single adult worm using Trizol was unsuccessful, NC: non computable. RNA from 100 worms could be extracted from Trizol and the *C_t_* value reflects per single adult.

Method	Sample	*C_t_*
Proteinase K + heat treatment	1 egg	25.6 ± 0.08
1 L1	25.2 ± 0.12
1 L2	22.4 ± 0.32
1 L3	21.9 ± 0.88
1 L4	19.8 ± 1.24
1 adult	18.9 ± 0.28
2 adults	18.0 ± 0.04
4 adults	16.8 ± 0.07
−RT	>35

Trizol	1 adult	NC
	100 adults	19.6 ± 0.10
